# The Inoculation for Cholera

**Published:** 1885-09-20

**Authors:** 


THE INOCULATION FOR CHOLERA, introduced by Dr. Ferran, of Valencia, Spain, has proved a disappointment, as theoretically it would seem it must do. Cholera is not a self-limited contagious disease, in which one attack protects against another ; therefore, the inoculation theory does not apply to this malady.

